# Targeting oncogenic fusion-driven NUT carcinoma with CRISPR-Cas9 genome editing

**DOI:** 10.1016/j.omton.2025.201068

**Published:** 2025-10-06

**Authors:** Maxim F. Carle, Tahereh Mohammadian Gol, Justin S. Antony, Alicia Roig-Merino, Mary E. Carter, Ulrich M. Lauer, Markus Mezger, Linus D. Kloker

**Affiliations:** 1 Virotherapy Center Tübingen (VCT), Department of Internal Medicine VIII, Medical Oncology and Pneumology, University Hospital, Tübingen, Germany; 2 Department of Hematology and Oncology, University Children Hospital, Tübingen, Germany; 3 MaxCyte Inc., Rockville, MD, USA

**Keywords:** MT: Regular Issue, CRISPR-Cas9, NUT carcinoma, BRD4::NUTM1 fusion, fusion driven cancer, cancer gene therapy, genome editing, BET inhibitor

## Abstract

NUT carcinoma (NC) is a highly aggressive malignancy characterized by an oncogenic fusion gene incorporating the *NUTM1* gene. To date, no established treatment options exist. CRISPR-Cas9 technology allows precise genomic targeting, thereby presenting a promising therapeutic strategy for cancers with well-defined genomic alterations such as oncogenic fusion genes. In this study, we investigated the effects of CRISPR-Cas9-mediated disruption of the *BRD4::NUTM1* fusion gene in NC cell lines using multiple single guide RNAs (sgRNAs) to target different sites of both fusion partner genes. Our experiments identified promising sgRNA candidates that were shown to successfully disrupt the *BRD4::NUTM1* fusion gene at the DNA level, thus leading to an efficient knockout of the aberrant fusion protein. This genetic disruption resulted in profound functional impairments in NC cells, which included a significant reduction in proliferative capacity, cell-cycle arrest, and induction of apoptosis. These findings underscore the dependency of NC on the *BRD4::NUTM1* fusion gene and highlight the potential of CRISPR-based strategies for targeting cancer at its genetic level. This approach also holds promise for the development of highly specific and effective therapies for many other oncogenic fusion driven cancers.

## Graphical abstract

**Figure:**
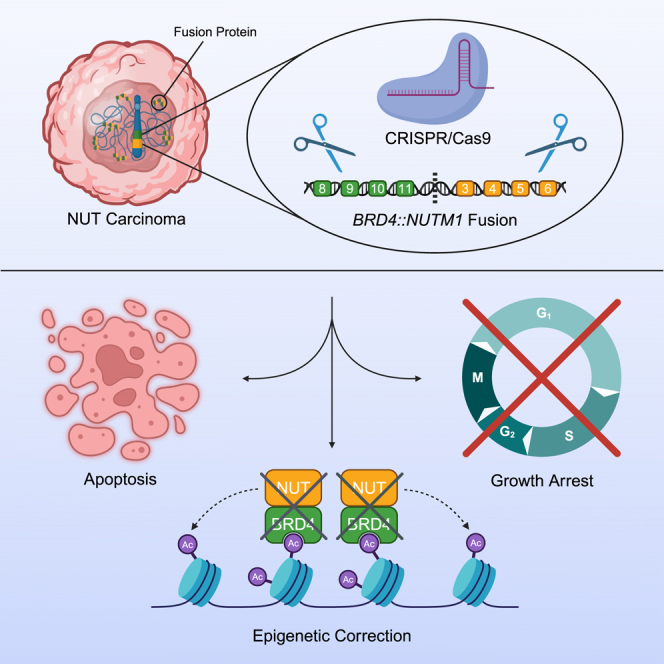

## Teaser

NUT carcinoma depends on the *BRD4::NUTM1* fusion, which current inhibitors can target but without durable clinical efficacy. This study demonstrates that CRISPR-Cas9 can directly disrupt this fusion, suppressing cancer cell growth. Therefore, cancer genome editing could be employed as an alternative and effective strategy for targeting fusion-driven solid cancers.

## Introduction

NUT carcinoma (NC) is a rare yet exceptionally aggressive malignancy characterized by a gene fusion involving *NUTM1* and a DNA binding fusion partner gene, most frequently *BRD4* and *BRD3*. The resulting fusion protein plays a key role in NC pathogenesis by reshaping the epigenetic landscape and driving the expression of oncogenic transcriptional programs.^1^^,^^2^^,^^3^ Despite current investigation of numerous treatments, no therapeutic option has proven to be effective so far. Surgery and conventional chemo- and radiotherapy remain the standard of care, albeit with only limited efficacy.^4^^,^^5^^,^^6^ Hence, the median survival of patients suffering from NC remains only six to seven months, thereby highlighting the urgent need for novel more effective treatment approaches.^7^

Experimental therapies include the use of bromodomain and extra-terminal domain inhibitors (BET) inhibitors, p300 inhibitors, EZH2 inhibitors or oncolytic viruses (OVs).^8^^,^^9^^,^^10^^,^^11^^,^^12^ Immune checkpoint inhibitors have also demonstrated therapeutic potential in single case reports and a case series.^13^^,^^14^ In the past, various BET inhibitors have demonstrated *in vitro* efficacy by targeting the common NUT fusion-partner proteins BRD4 and BRD3, but their clinical success has been limited.^8^^,^^15^ Given that the expression of NUT is usually restricted to spermatogenic cells, NUT represents a potential therapeutic target in NC.^16^ Since no molecular inhibitors of NUT have been described so far, an alternative is to target NUT at a genomic level in NC. Previous research has demonstrated that suppressing the *BRD4::NUTM1* fusion protein using RNA interference and proteolysis targeting chimeras can significantly reduce NC cell proliferation.^17^^,^^18^^,^^19^ However, these methods are inherently reversible, whereas directly targeting NUT at the DNA level could provide a more durable and lasting solution to halt cancer progression.

CRISPR (clustered regularly interspaced short palindromic repeats) genome editing has emerged as a powerful tool for precise DNA manipulation. Although CRISPR-based therapies have progressed to clinical applications exemplified by the European Medical Agency and the United States Food and Drug Administration approval of the first CRISPR therapy, exagamglogene autotemcel (Casgevy)—their efficient use in oncology and precise intratumoral delivery remain challenging.^20^ Previous studies have demonstrated that CRISPR-mediated DNA cleavage can selectively kill cancer cells by targeting tumor-specific DNA sequences.^21^^,^^22^ Similar antiproliferative effects have been reported in other fusion driven cancers using fusion gene specific targeting systems, including *BCR::ABL*-driven CML, *RUNX1::RUNX1T1*-driven AML, and Ewing sarcoma.^23^^,^^24^^,^^25^

In this study, we assessed the feasibility of using CRISPR-Cas9 to specifically target and disrupt the *BRD4::NUTM1* fusion gene in NC cells, thereby inhibiting the oncogenic process driven by this aberrant fusion. To achieve this, we designed and tested the efficacy of a panel of Cas9 single guide ribonucleic acids (sgRNAs) directed at exons of *BRD4* and *NUTM1* both within and outside the fusion gene. We evaluated DNA editing, protein knockout, and proliferation arrest in two *in vitro* NC models: The NC cell line HCC2429, which harbors a fusion of *BRD4* exon 11 with *NUTM1* exon 3, and the NC cell line 143100, in which *BRD4* exon 14 is fused with *NUTM1* exon 3.^17^^,^^26^

## Results

### CRISPR targeting strategy and sgRNA design

**Figure 1:**
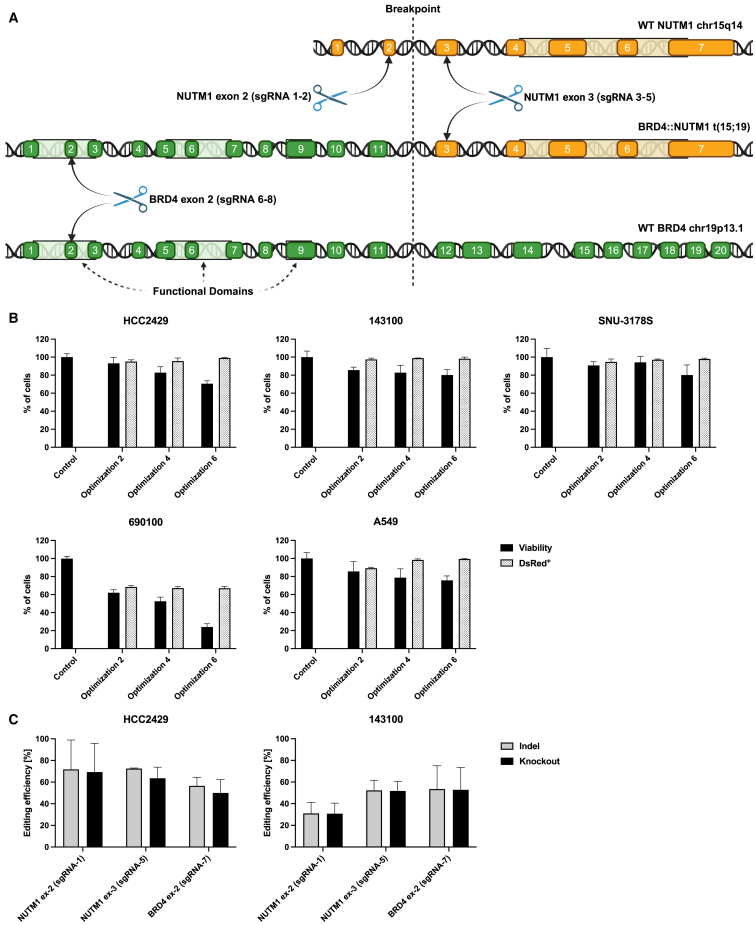
CRISPR-Cas9 targeting of the oncogenic *BRD4::NUTM1* fusion gene (A) Schematic representation of the CRISPR-Cas9 strategy to disrupt the *BRD4::NUTM1* fusion gene. The *BRD4::NUTM1* fusion gene of NC cell line HCC2429 is shown. Cas9 guided by sgRNAs targeting *NUTM1* exon 3 or *BRD4* exon 2 can disrupt the fusion gene, whereas sgRNA directed at *NUTM1* exon 2 (not part of the fusion) only disrupts the wildtype allele of NUTM1, serving as a control. (B) Optimization of electroporation settings on the MaxCyte ExPERT GTx electroporator. Cells from NC cell lines HCC2429, 143100, SNU3187S, 690100 and from NSCLC cell line A549 (control) were transfected with dsRed mRNA and transfection efficiencies were evaluated at 24 h post transfection (hpt) using flow cytometry. Cell viabilities were measured employing the MTS assay. Error bars represent mean (SD) of two independent experiments. (C) Gene editing efficacy in NC cell lines HCC2429 and 141300. Indel and knockout scores were calculated at 72 hpt with Cas9 RNP and the most effective sgRNAs. Error bars represent mean (SD) of four independent experiments.

For our CRISPR-Cas9 editing experiments, we targeted three different exons to achieve gene knockout by inducing indels: two within the *BRD4::NUTM1* fusion and one outside of it (Figure 1A). Specifically, we designed two sgRNAs targeting *NUTM1* exon 2 (outside of the gene fusion), which served as a negative control only targeting wild-type (WT) *NUTM1*, and three sgRNAs each for *NUTM1* exon 3 and *BRD4* exon 2, both gene loci within the fusion gene of the NC cells and therefore able to achieve its disruption. Early exons were prioritized to achieve a frameshift with the highest likelihood.

### Electroporation of NUT carcinoma cells

To achieve efficient transfection of the Cas9 RNP, we first determined appropriate electroporation settings using the MaxCyte ExPERT GTx electroporator. Four NC cell lines and A549 non-small cell lung cancer (NSCLC) control cells were electroporated with mRNA encoding dsRed using three different electroporation settings (optimization 2, 4, and 6), and transfection rates were measured via flow cytometry, while cellular viability was assessed using MTS assays. Transfection efficiencies and cell viabilities vary based on the optimization protocol used (Figure 1B). Among the cell lines tested, NC cell lines HCC2429, 143100, and SNU-3178S as well as the NSCLC cell line A549 demonstrated transfection rates exceeding 95% for dsRed, thus making them suitable for subsequent experiments. In contrast, the NC cell line 690100 was less amenable to electroporation, and we achieved only a 67% transfection rate with lower cell viability. We selected the electroporation program optimization 4 for all cell lines in future experiments as it provided high transfection efficiencies necessary for minimizing the impact of non-transfected cells on the results of our experiments, while maintaining adequate cell viability.

### Optimization of different sgRNAs

To test sgRNA and Cas9 efficacy for each locus to induce indels and knockouts using our experimental setup, we first transfected A549 cells with Cas9 and the eight selected sgRNAs. Sanger sequencing 72 h after gene editing revealed modest differences in indel scores (17% range for *NUTM1* exon 2, 17% for *NUTM1* exon 3, and 8% for *BRD4* exon 2) but large differences in knockout scores (12% for *NUTM1* exon 2, 51% for *NUTM1* exon 3, and 30% for BRD4 exon 2) (Figure S1). The observed discrepancy between indel and knockout score can be explained by a large proportion of indels being multiples of three bases, therefore not causing frameshift mutations. Overall, satisfactory indel and knockout efficiency was achieved for each target locus and the sgRNAs *NUTM1* ex-2 sgRNA-1 (indel 71%, knockout 66%), *NUTM1* ex-3 sgRNA-5 (indel 93%, knockout 92%), and *BRD4* ex-2 sgRNA-7 (indel 85%, knockout 81%) were selected for subsequent experiments.

After this initial screening of sgRNAs, we transfected NC cells HCC2429 and 143100 with Cas9 and the most promising sgRNAs for each target gene. Sanger sequencing at 72 hours post-transfection (hpt) confirmed effective gene editing through the formation of indels and knockouts, albeit at lower frequencies than observed in A549 cells (mean indel scores: 57%–73% in NC cell line HCC2429 and 31%–54% in NC cell line 143100) (Figure 1C). SNU3178S were not subjected to gene editing since they harbor a *BRD3::NUTM1* fusion between *BRD3* exon 10 and *NUTM1* exon 6, therefore necessitating different sgRNAs from our panel.

### Proliferation and viability assays

**Figure 2:**
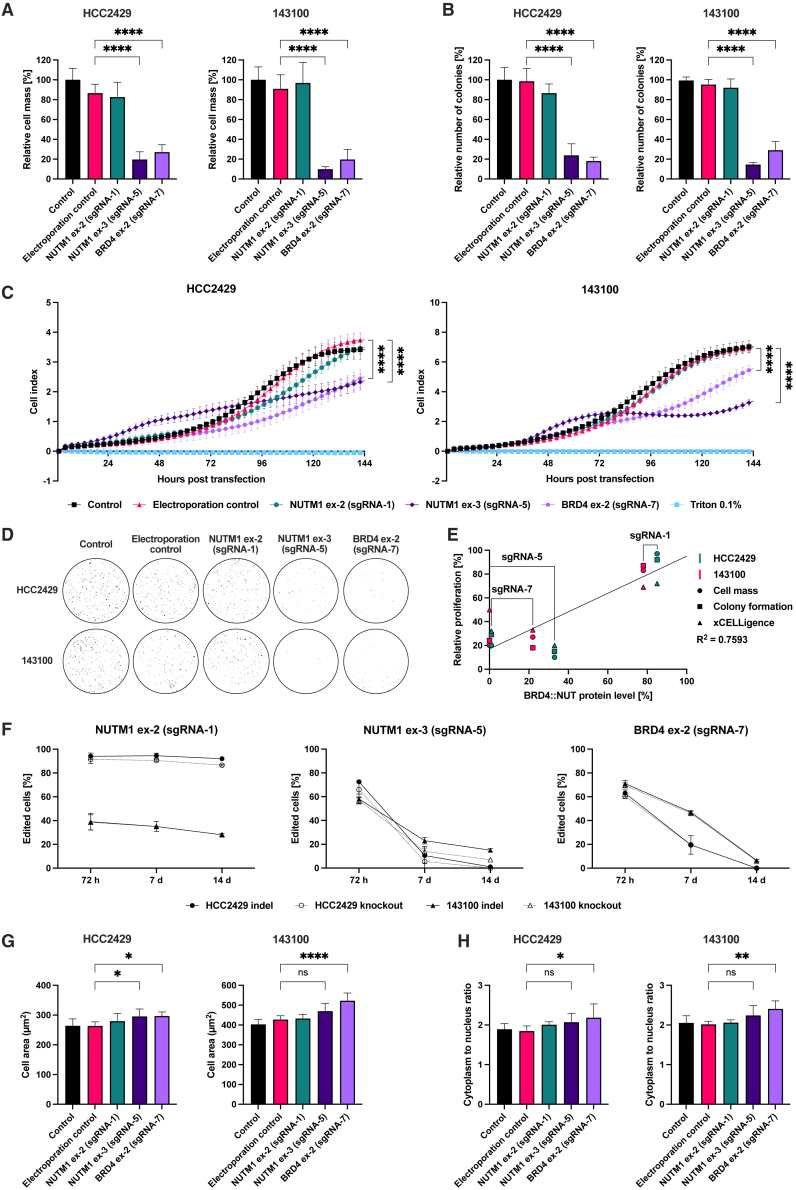
CRISPR-Cas9 disruption of *BRD4::NUTM1* inhibits proliferation of NC cells (A) Reduction in tumor cell masses after gene editing. Sulforhodamine B (SRB) viability assays were performed at 96 hpt employing NC cell lines HCC2429 and 143100. Mean (SD) from two independent experiments performed in six technical replicates are shown. (B) Colony formation assay after gene editing. 1,000 cells from NC cell lines HCC2429 and 143100 were seeded at low densities directly after transfection with Cas9 RNP and different sgRNAs and allowed to grow for one week. Mean (SD) from two independent experiments performed in triplicates are shown. (C) Real-time tumor cell growth monitoring using the xCELLigence assay. Electrical impedance was recorded continuously for 144 hpt on NC cell lines HCC2429 and 143100; measurements were performed every 30 min. Shown are mean (SD) from one representative result of two independent experiments. (D) Images of colony formation assay. Representative images of cell colonies stained with crystal violet eight days after seeding cells from NC cell line HCC2429 and six days for NC cell line 143100. (E) Correlation between BRD4::NUT protein levels and NC cell proliferation. Each data point corresponds to transfection with one sgRNA, plotting the BRD4::NUT protein level (measured by western blotting) against a functional proliferation readout (SRB, colony formation, or xCELLigence assay). Points include results from both NC cell lines HCC2429 and 143100. (F) Gene editing kinetics in NC cell lines HCC2429 and 143100. Indel and knockout scores were measured over 14 days following gene editing with three different sgRNAs targeting *NUTM1* exon 2 (sgRNA-1), *NUTM1* exon 3 (sgRNA-5), and *BRD4* exon 2 (sgRNA-7). Error bars represent mean (SD) of two independent experiments. (G) Cell area of NC cell lines HCC2429 and 143100 after gene editing. Immunofluorescence microscopy images stained for β-actin and DAPI were acquired 72 hpt and used to measure single cell areas. Shown are mean (SD) of two independent experiments. (H) Cytoplasm to nucleus ratio in NC cell lines HCC2429 and 143100. Images of cells stained for β-actin and DAPI were used to calculate the ratio of cytoplasmic to nuclear area. Shown are mean (SD) of two independent experiments. ∗*p* < 0.05, ∗∗*p* < 0.01, and ∗∗∗∗*p* < 0.0001.

Three different assays were performed to assess changes in proliferation and viability of NC cells after gene editing. SRB assays conducted at 96 hpt showed a large reduction in total protein mass following gene editing at the *BRD4::NUTM1* fusion site (Figure 2A). Specifically, gene editing at *NUTM1* exon 3 reduced cell mass to 19.6% ± 7.9% in HCC2429 (*p* < 0.0001) and 9.9% ± 2.6% in 143100 cells, while editing at *BRD4* exon 2 reduced cell mass to 27.0% ± 7.5% in HCC2429 (*p* < 0.0001) and 19.6% ± 10.2% in 143100 (*p* < 0.0001). As expected, electroporation control without Cas9 RNP and editing of WT NUTM1 by targeting NUTM1 exon 2 served as a control, and showed no significant changes in cell mass beyond the expected effects of electroporation.

Colony formation assays further demonstrated effective reduction in the proliferative capacity of NC cells after gene editing at *NUTM1* exon 3 (23.8% ± 11.8% in HCC2429 and 14.5% ± 2.1% in 143100, *p* < 0.0001 for both) and *BRD4* exon 2 (18.0% ± 4.0% in HCC2429 and 19.0% ± 8.6% in 143100, *p* < 0.0001 for both) (Figure 2B). As expected, no significant reduction in colony formation was observed in the negative control groups or after WT NUT editing.

Continuous real-time monitoring of cell growth by cell impedance using the xCELLigence system over 6 days revealed close alignment of growth curves between untreated control cells, electroporation controls, and cells edited at *NUTM1* exon 2, for both NC cell lines HCC2429 and 143100 (Figure 2C). Cells edited at *NUTM1* exon 3 exhibited a distinct growth pattern, initially showing an unexpected transient increase in cell index during the first 72 hpt. Following this initial phase, the two NC cell lines showed different behavior, while HCC2429 cells continued to grow in a linear fashion, 143100 cells exhibited a transient stagnation before resuming proliferation toward the end of the recording. In contrast, gene editing at *BRD4* exon 2 showed no initial increase in cell index in either NC cell line and resulted in slower growth compared with control groups. After 144 h, the cell index of HCC2429 and 143100 was significantly reduced following gene editing at *NUTM1* exon 3 and *BRD4* exon 2 (*p* < 0.0001 for all).

Images of stained colonies of HCC2429 and 143100 corroborated these findings from quantitative assays, showing a drastic decrease in colony number after gene editing at *NUTM1* exon 3 and *BRD4* exon 2 with no comparable change in electroporation controls and after gene editing at *NUTM1* exon 2, thus indicating the specific efficacy of the sgRNAs used (Figure 2D).

Additionally, to link protein knockdown with functional outcomes, we performed a correlation analysis comparing the residual *BRD4::NUTM1* protein (quantified through western blotting) to the observed changes in proliferation 72 hpt in SRB and colony formation assays and the slope xCELLigence growth curves (Figure 2E). NC cell lines HCC2429 and 143100 which were transfected with Cas9 and each sgRNA were plotted for their residual *BRD4::NUTM1* levels against the corresponding proliferative readout. This analysis revealed a strong direct correlation (R^2^ = 0.7593), with a slope of 0.7756 and an intercept of 16.94, thereby indicating that reduction in *BRD4::NUTM1* consistently aligns with pronounced anti-proliferative effects in NC cells.

To evaluate the stability of gene editing over time, indel and knockout scores were measured over 14 days in HCC2429 and 143100, starting at 72 hpt (Figure 2F). After gene editing at *NUTM1* exon 2, mean indel scores declined only by an absolute 2% in HCC2429 and 11% in 143100. In contrast, mean indel scores following gene editing at *NUTM1* exon 3 resulted in a strong loss from 73% to 1% in HCC2429 and from 58% to 15% in 143100. Likewise, indel scores after gene editing at *BRD4* exon 2 decreased from 63% to 0% in HCC2429 and from 71% to 7% in 143100. The proportion of knockout-inducing indels among all indels decreased over time when editing *NUTM1* exon 3, from a mean of 91% at 72 h to 42% after 7 days and 0% after 14 days.

To detect any change in cell size, immunofluorescence microscopy with anti-β-actin staining was performed at 72 hpt (Figure 2G). Quantification of cell area after gene editing at *NUTM1* exon 3 revealed a 12.1% increase in mean cell area in HCC2429 (*p* = 0.0477) and a 9.9% increase in 143100 (*p* = 0.0653). Following gene editing at *BRD4* exon 2, mean cell area increased by 12.6% in HCC2429 (*p* = 0.0372) and by 22.3% in 143100 (*p* < 0.0001).

To assess proportional changes in cell morphology, the measured cellular area was related to the corresponding nuclear area, determined from DAPI staining in the same experiment, and used to calculate cytoplasm-to-nucleus ratios in both cell lines (Figure 2H). Gene editing at NUTM1 exon 3 increased the ratio by 11.9% in HCC2429 (*p* = 0.2200) and 10.9% in 143100 (*p* = 0.0942). Gene editing at *BRD4* exon 2 increased the ratio by 17.8% in HCC2429 (*p* = 0.0310) and 19.3% in 143100 (*p* = 0.0020).

While cells showed a slight upward shift in mean cell area and cytoplasm to nucleus ratio following gene editing at *NUTM1* exon 2, these changes were not significant compared with the electroporation control (Figures 2G and 2H).

### Validation of *BRD4::NUT* fusion protein knockout

In order to confirm whether gene editing of the *BRD4::NUTM1* fusion on a genome level translated into knockout on the protein level, we performed western blots to detect the NUT fusion protein and c-Myc, a key oncogene upregulated in NC. Samples were collected immediately following transfection, at 24, 48, and 72 hpt. Vinculin was used for protein normalization, and protein samples from A549 cells (with NSCLC origin) served as a general negative control for NUT.

**Figure 3:**
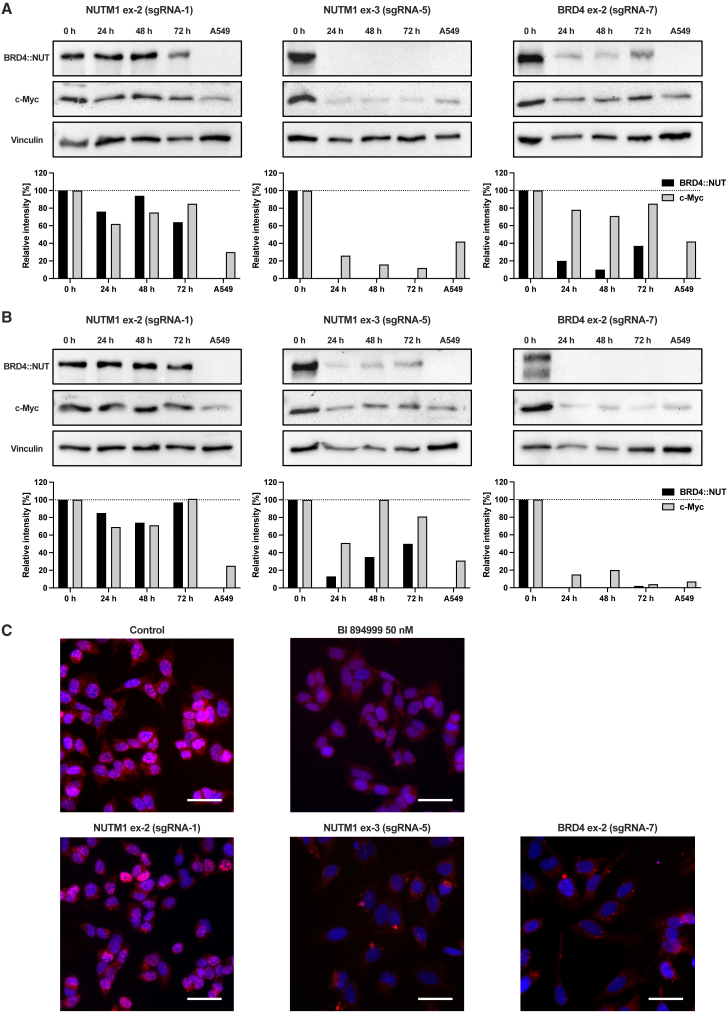
*BRD4::NUTM1* fusion oncogene disruption achieves depletion of the BRD4::NUT fusion protein (A) BRD4::NUT knockout in NC cell line HCC2429. Western blots of cells transfected with Cas9 RNP and sgRNAs targeting *NUTM1* exon 2, *NUTM1* exon 3, and *BRD4* exon 2, starting at 0 hpt. Untreated cells from NSCLC cell line A549 served as a negative control for BRD4::NUT. Graphs show densitometric quantification of band intensity of BRD4::NUT fusion protein and c-Myc after normalization to vinculin (loading control). (B) BRD4::NUT knockout in NC cell line 143100. Western blots of cells transfected with Cas9 RNP and sgRNAs targeting *NUTM1* exon 2, *NUTM1* exon 3, and *BRD4* exon 2, starting at 0 hpt. Untreated cells from NSCLC cell line A549 served as a negative control for BRD4::NUT. Graphs show densitometric quantification of band intensity of BRD4::NUT fusion protein and c-Myc after normalization to vinculin (loading control). (C) Immunofluorescence analysis of BRD4::NUT. Cells from NC cell line 143100 were transfected with Cas9 RNP and sgRNAs targeting *NUTM1* exon 2, *NUTM1* exon 3, and *BRD4* exon 2. Merged IF images of NUT and DAPI were taken at 72 hpt. Untreated (control) and BET inhibitor (BI984999, 50 nM) treated cells served as references for distribution of NUT protein. Scale bars, 50 μm.

In HCC2429 cells, gene editing targeting *NUTM1* exon 2 showed little effect on the NUT fusion protein and c-Myc levels (Figure 3A). NUT WT (120 kDa) was not detectable (Figure S2). Gene editing at *NUTM1* exon 3 resulted in complete elimination of the NUT fusion protein and a marked reduction of c-Myc (to 26% at 24 hpt). Gene editing targeting *BRD4* exon 2 led to a small reduction in BRD4::NUT (to 20% at 24 hpt), and only a slight decrease in c-Myc once normalized to vinculin. Expectedly, A549 cells were found to be negative for NUT WT (120 kDa) and NUT fusion protein (180–220 kDa) in all experiments.

In 143100 cells, gene editing at *NUTM1* exon 2 showed no relevant effect on NUT fusion protein and c-Myc levels (Figure 3B). NUT WT (120 kDa) was not detectable (Figure S3). Gene editing at *NUTM1* exon 3 led to a strong reduction in NUT fusion protein levels (to 10% at 24 hpt) and c-Myc (to 50% at 24 hpt). From 48 hpt and 72 hpt, samples showed an increase in BRD4::NUT and c-Myc levels over time. Gene editing at *BRD4* exon 2 achieved complete loss of the NUT fusion protein and a large decrease in c-Myc (to 15% at 24 hpt).

Next, we performed immunofluorescence microscopy to further analyze NUT fusion protein localization and quantity. The untreated control and cells after knockout of WT NUT by targeting *NUTM1* exon 2 exhibited the characteristic speckled nuclear staining of NUT, which resulted from NUT mega-domains within nuclei of NC cells (Figure 3C). Treatment of NC cells with the BET inhibitor BI894999 caused this characteristic speckling to dissolve into a homogenous staining of the nucleus by detachment of the NUT fusion protein after blockage of the acetylated histone binding domains of BRD4.^27^ In contrast, knockout of *BRD4::NUTM1* through gene editing at *NUTM1* exon 3 and *BRD4* exon 2 achieved total elimination of NUT protein from the nucleus, thereby leaving only DAPI-stained nuclei visible. Additionally, NC cells exhibited a modest increase in size after gene editing, which likely reflected changes in cell morphology following fusion gene knockout.

### Cell cycle and apoptosis analysis

**Figure 4:**
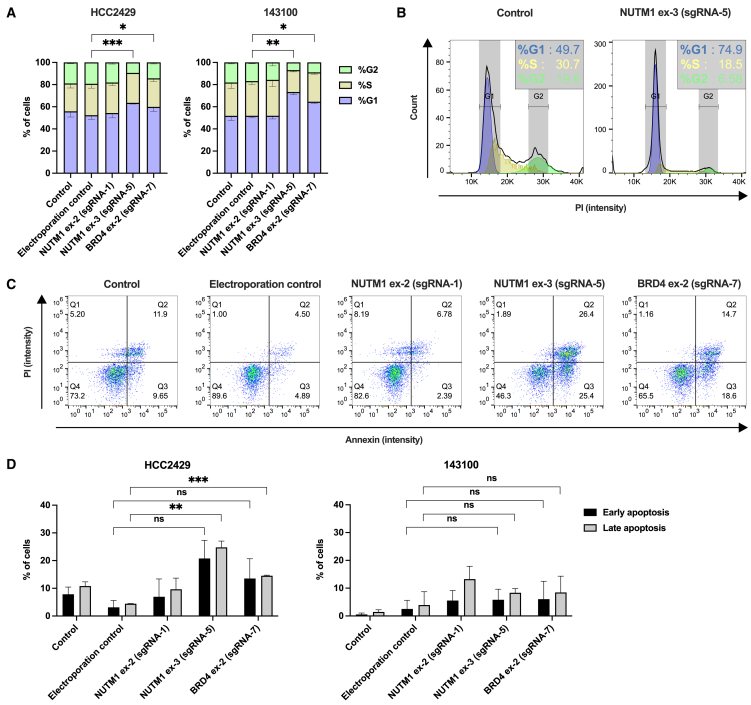
*BRD4::NUTM1* disruption induces cell-cycle arrest and apoptosis (A) Cell cycle analysis in NC cell lines HCC2429 and 143100. Cells were stained at 72 hpt using PI and analyzed with flow cytometry. Mean (SD) of two independent experiments are shown, with unpaired *t* tests performed for significance. (B) Cell cycle histogram of NC cell line 143100. Comparison of untreated (control) cells and cells at 72 hpt with Cas9 RNP and *NUTM1* exon 3. Fraction of cells in each phase of the cell cycle were measured using PI staining and flow cytometry. (C) Apoptotic fractions of NC cell line HCC2429. Cells were stained with annexin V and PI at 72 hpt, and analyzed by flow cytometry. Representative plots from two independent experiments are shown. (D) Analysis of apoptotic cells of NC cell lines HCC2429 and 143100. Cells were stained with annexin V and PI at 72 hpt, and analyzed by flow cytometry. Annexin^+^/PI^−^ cells are classified as early apoptotic, and annexin^+^/PI^+^ cells as late apoptotic. Data shown represent mean (SD) of two independent experiments, with unpaired *t* tests performed for significance. ∗*p* < 0.05, ∗∗*p* < 0.01, and ∗∗∗*p* < 0.005.

To better understand the mechanisms underlying the observed anti-proliferative effect after gene editing, we performed cell cycle analyses and quantified markers for apoptosis. Cell-cycle analysis via intracellular PI staining at 72 hpt revealed a clear G1-arrest in both NC cell lines tested (Figure 4A). In HCC2429, the G2 fraction decreased significantly from 19.3% ± 0.3% in the electroporation control to 9.4% ± 0.4% (*p* = 0.0011) after gene editing at *NUTM1* exon 3 and to 14.4% ± 1.3% (*p* = 0.0364) after gene editing at *BRD4* exon 2. In 143100 cells, the G2 fraction decreased from 15.6% ± 3.0% in the electroporation control to 7.0% ± 0.8% (*p* = 0.0094) after gene editing at *NUTM1* exon 3 and to 9.0% ± 0.1% (*p* = 0.0100) after gene editing at *BRD4* exon 2.

This cell-cycle arrest became visually evident in the histograms of PI-stained 143100 cells (Figure 4B), where the G1 peak (blue) was noticeably elevated and the G2 peak (green) was diminished after gene editing at *NUTM1* exon 3. The graphs further illustrate the change of G1, S, and G2 fractions, which were calculated using the Watson pragmatic algorithm.

Apoptosis was analyzed by flow cytometry of cells stained for annexin V and PI. In HCC2429 cells, scatterplots indicate the emergence of a distinct annexin^+^/PI^−^ population and a double-positive population after gene editing of the *BRD4::NUTM1* fusion gene (Figure 4C).

Apoptosis was quantified by defining early apoptotic cells as annexin^+^/PI^−^ and late apoptotic cells as annexin^+^/PI^+^ (Figure 4D). In HCC2429, gene editing increased the mean apoptotic fraction of cells when comparing the electroporation control group (3.2% ± 1.8% in early apoptosis and 4.5% ± 0.1% in late apoptosis) with cells following gene editing at *NUTM1* exon 3 (20.8% ± 4.6% in early apoptosis, *p* = 0.0667 and 24.8% ± 1.6% in late apoptosis, *p* = 0.0061) and after gene editing at *BRD4* exon 2 (13.6% ± 5.1% in early apoptosis, *p* = 0.1911 and 14.6% ± 0.2% in late apoptosis, *p* = 0.0002). In 143100, the mean apoptotic fraction did not change significantly after gene editing at *NUTM1* exon 3 and *BRD4* exon 2 relative to the electroporation control.

### Off-target analysis

**Figure 5:**
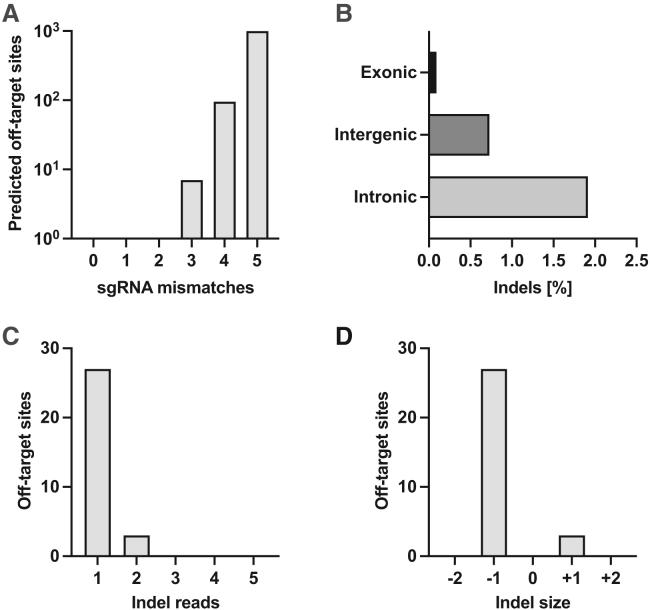
Off-target analysis (A) *In silico* off-target prediction. Potential off-target sites for *NUTM1* ex-3 (sgRNA-5) were predicted *in silico*, allowing up to five base mismatches between sgRNA and DNA. (B) Number and distribution of off-target edits. Whole genome sequencing was performed on HEK293 cells transfected with Cas9 RNP and *NUTM1* ex-3 (sgRNA-5) and aligned to the hg38 reference genome to analyze predicted sites for indels. Detected indels at analyzed off-target sites are shown. (C) Number of reads supporting each off-target indel. The count of reads carrying indels in HEK293 cells at each potential off-target site is shown. Mean reads per locus were 45. (D) Indel size distribution. The size and frequency of indels detected in HEK293 cells at analyzed off-target sites are shown.

To detect possible off-target activity of Cas9 in combination with *NUTM1* ex-3 sgRNA-7, we used *in silico* prediction of the most likely off-target sites by CasOFFinder, allowing up to five mismatches between the sgRNA and target DNA (Figure 5A). No off-target sites were predicted for zero, one or two base mismatches to the sgRNA. Allowing three base mismatches, seven potential off-target sites were identified, whereas 95 and 996 potential off-target sites were computed for four and five base mismatches, respectively. Next, we performed WGS on HEK293 cells treated with NUTM1 ex-3 sgRNA-7 at 72 hpt and analyzed the 1,098 predicted off-target sites for indels. We selected HEK293 cells since they resemble healthy human cells more closely, where off-target effects are a much greater concern compared with cancer cells. No indels were identified at predicted off-target sites with three mismatches to the sgRNA. A total of 30 indels were found at sites with four or five mismatches to sgRNA, without any apparent clustering or genomic preference (Figure 5B). All detected indels are located in non-coding regions—either intronic or intergenic—with the exception of a single deletion observed in the 3′-UTR of the *SPATA33* gene (Table S1). Notably, two of these sites exhibited a relative allele frequency of 100%, thereby indicating that they are most likely pre-existing genetic variants. Of the remaining 28 sites containing indels, three had more than one read supporting the variant, thus suggesting a high level of confidence that these are true off-target hits (Figure 5C). The remaining 25 indels were only detected by a single read, which potentially emerged from inaccuracy of DNA sequencing or single nucleotide variants (SNVs) in cell culture. Analysis of individual reads revealed that 27 off-target sites exhibited a −1 deletion, while the remaining three sites showed a +1 insertion (Figure 5D).

## Discussion

Our data show that CRISPR-Cas9-mediated disruption of the *BRD4::NUTM1* fusion gene effectively reduces the proliferation of NC cells. sgRNAs targeting different exons of *BRD4* and *NUTM1* achieved efficient genomic editing and consequent knockout of the fusion protein, thus confirming the expected mechanism behind the observed functional change. Proliferation assays revealed a strong reduction in NC cell proliferation after gene editing, which is attributable to increased apoptosis and cell-cycle arrest. However, we also observed that not all cells were successfully edited, and that the fraction of cells with disrupted *BRD4::NUTM1* drops near zero within 14 days. Although we cannot completely exclude that some NC cells adapted to loss of *BRD4::NUTM1*, our experiments showed that any non-edited NC cells quickly outgrew the edited cells. Due to the fast selection of non-edited cells, more advanced, longitudinal single-cell analyses may detect any existing escape mechanisms. Cells with function-maintaining indels appeared to persist slightly longer than knockout cells, but their frequency also decreased over time. This underscores the challenge of achieving complete gene disruption with a single-time gene editing strategy. Interestingly, gene editing at *NUTM1* exon 2, which lies outside the fusion gene, showed no functional change in NC cells. Longitudinal tracking of cells with disrupted WT *NUTM1* showed only a small decrease of this edited fraction over time, indicating no relevant effect on proliferation rates compared with non-edited NC cells. Together, these results suggest that WT *NUTM1* is not involved in the pathomechanism of NC. Furthermore, NC cell line HCC2429 entered apoptosis preferentially to cell-cycle arrest, compared with 143100, which may be explained by a mutated p53/pRB axis in HCC2429.^26^

The observed changes in NC cells following *BRD4::NUTM1* knockout can be traced to the loss of the aberrant NUT fusion protein, which drives the unrestrained hyperacetylation underlying NC pathogenesis. This is further supported by the significant reduction in c-Myc levels, a key downstream target upregulated by the aberrant NUT fusion protein that sustains proliferation. Increased apoptosis, growth arrest, and altered cell morphology, primarily increased cell size, likely reflect the recovery of cellular control and squamous differentiation that have been previously reported following disruption of *BRD4::NUTM1* activity.^9^^,^^28^^,^^29^^,^^30^ The transient increase in cell index observed during xCELLigence analysis following gene editing at *NUTM1* exon 3 was potentially caused by this cellular dilation rather than increased proliferation. Our findings align with previous research that implicates the *NUTM1* fusion gene as the key driver of NC oncogenesis, as evidenced by the induction of NC *in vivo* through activation of the *BRD4::NUTM1* fusion gene and growth arrest following fusion protein knockdown or degradation.^2^^,^^3^^,^^17^^,^^18^^,^^19^ Notably, a recently published patent on CRISPR-Cas9-mediated gene editing of *NUTM1* shows primarily reduced growth rates and increased apoptosis after targeting different gene sequences and exons of *NUTM1*, thereby adding independent validation to our results.^31^

In oncology, CRISPR-based therapies offer a novel approach for targeting previously inaccessible drivers of cancer. Studies utilizing CRISPR-Cas9 to disrupt oncogenic fusion genes in other fusion-driven cancers yielded results similar to our findings for NC: In AML and CML, Cas9-mediated disruption of fusion genes significantly reduced proliferation both *in vitro* and *in vivo*.^23^^,^^24^ In Ewing sarcoma, *in vitro* analyses likewise demonstrated decreased proliferation and cell-cycle arrest, and additionally revealed extensive senescence following fusion gene loss.^25^ Further, *in vivo* delivery of an *EWSR1::FLI1* disrupting dual intron targeting system led to lower Ki67 proliferative index, higher levels of cleaved caspase-3 and prolonged survival in an Ewing sarcoma mouse model.^32^

A key advantage of performing a fusion gene knockout in NC is that *NUTM1* expression is largely restricted to the testes, making a widespread knockout of *NUTM1* tolerable *in vivo*.^16^ In contrast, *BRD4* is ubiquitously expressed and plays an essential role in transcriptional regulation by modulating RNA polymerase II activity.^33^^,^^34^ Its widespread knockout would most likely prove detrimental, as *in vivo* silencing of BRD4 has been shown to severely damage various rapidly proliferating tissues, primarily blood, skin, and the intestine.^35^ An alternative approach intended to circumvent this issue uses dual-sgRNA targeting of two introns flanking the breakpoint region to achieve an exon excision and frameshift in the oncogene.^24^^,^^32^ However, since this requires efficient Cas9 cleavage at two loci followed by excision of the desired exon, net excision rates are reportedly much lower than knockout with one exon-targeting sgRNA.^36^ For these reasons, our off-target analysis prioritized the more promising *NUTM1* ex-3 sgRNA-5, which demonstrated both high specificity and efficacy.

For most CRISPR-based therapies, safety is generally a key concern, which typically brings interest in studying specificity. While research on CRISPR-Cas9 and its derivatives highlight specificity compared to other genetic modifiers, they are not entirely error-free and often exhibit some unintended activity in undesired regions.^37^^,^^38^^,^^39^^,^^40^^,^^41^ A variety of methods exist for off-target analysis, each with its own advantages and limitations, but no single approach has emerged as the definitive standard.^42^^,^^43^^,^^44^ In this study, we relied on WGS for the ability to analyze and prioritize potential off-target sites based on predicted likelihood. While any method for off-target analysis carries a risk of false positives, our results demonstrate that Cas9, in combination with our optimized sgRNA, likely exhibits some off-target activity at very low frequencies.

Off-target analysis was performed in HEK293, whose low mutational burden and well-studied genome minimize the risk of false positives. Since no untreated HEK293 cells were sequenced as a reference, the interpretation relies on including only indels with low variant allele frequencies (VAFs) while indels with 100% VAF are considered pre-existing variants. Unlike other sequencing methods that selectively capture double-strand breaks (i.e., GUIDE-Seq), this unbiased WGS approach only detects resulting indels, thereby adding an additional level of uncertainty. Notably, all detected off-target edits occurred in non-essential or functionally irrelevant regions. None of the detected off-target sites were located in the open reading frame or essential regulatory regions, thereby making further oncogenic events unlikely. For indels detected within only a single read, conclusions on true Cas9 off-target activity have to be made with caution, as they can also arise from inaccuracy in DNA sequencing or from serial passaging in cell culture. Sequencing typically introduces an indel error rate of approximately 10^−5^, two orders of magnitude below the measured off-target indel frequency of 10^−3^ for bases analyzed, thus indicating that the observed edits likely represent bona fide Cas9 activity rather than random sequencing errors.^45^ Another large risk stems from new chromosomal rearrangements in healthy cells involving *NUTM1* and generating new oncogenes, caused by double-strand breaks from Cas9 cleavage. Since the approach chosen in this study is not able to detect translocations or novel fusions, future studies would need further investigation. However, in light of the aggressive nature and poor prognosis of NC this risk is unlikely to outweigh potential therapeutic benefits.

Electroporation is currently the preferred delivery method for *ex vivo* CRISPR-based gene therapies due to its relative simplicity and reproducibility.^46^^,^^47^ While we relied on electroporation in this study, it should be noted that electroporation typically does not achieve 100% transfection efficiency, and the residual untransfected NC cells may regrow over time, thereby potentially skewing experimental results.^48^ Furthermore, effective *in vivo* delivery of Cas9 and sgRNAs to directly target solid tumors remains a significant challenge. To date, viral vectors and lipid nanoparticles show the most promise for *in vivo* delivery.^49^^,^^50^^,^^51^ CRISPR-Cas9 may also be used to enhance oncolysis of existing OVs by targeting tumor-driving genes. For instance, CRISPR-Cas9-armed HSV has shown efficacy in HPV16-positive cervical cancer by disrupting viral oncogenes.^52^ In NC, future studies could explore intratumoral delivery of CRISPR-Cas9 via an OV as a novel enhancement of virotherapy through localized genome editing of the aberrant *NUTM1* fusion.

As all experiments were performed in cell lines, validation in primary NC cells and a mouse model will be an important next step toward translation. Looking forward, our validated sgRNAs open the door to advanced genetic therapies for NC. The DNA double-strand breaks induced by CRISPR-Cas9 could also enable the integration of new therapeutic genes, thus potentially enhancing lytic effects or stimulating a tumor-specific immune response. This concept has been proven possible by the successful integration of a HSV1-tk suicide gene into different cancerous fusion genes, thereby achieving cell-specific apoptosis and reduced tumor burden.^53^ Alternatively, utilizing sgRNAs that specifically target the unique breakpoint of the NUTM1 fusion gene could provide exceptional specificity while necessitating customized sgRNA designs for individual patients, due to the variable nature of fusion breakpoints. Advanced CRISPR-based gene editing technologies such as base editor or prime editor make many more approaches conceivable that could enable gene disruption with lower risks from off-target editing or enable new ways for targeting NC cells.

## Materials and methods

### Cell culture

NC cell lines HCC2429, 143100, and 690100 were kindly provided by Prof. Jens Siveke from University Hospital Essen, Germany. The NC cell line SNU-3178S was kindly provided by Prof. Young Seok Ju from Korea Advanced Institute of Science and Technology, Daejeon, Korea. Cell lines A549 and HEK293 were obtained from the American Type Culture Collection (Manassas, VA, USA). All cells were maintained in Dulbecco’s modified Eagle’s medium (DMEM), supplemented with 10% fetal bovine serum (FBS).

### Electroporation

sgRNAs were designed to maximize on-target activity while minimizing off-target effects, based on the optimized scoring algorithm described by Doench et al.^54^ sgRNAs were generated using CHOPCHOP software v.3,^55^ the Synthego CRISPR Knockout Design Tool IDT CRISPR-Cas9 guide RNA design checker and the Stemcell ArciTect sgRNA design tool. For each locus, sgRNAs were ranked by predicted activity score and off-target profile and the top two to three candidates selected. The exact sequences used are listed in Table 1. Cells were electroporated using the MaxCyte ExPERT GTx electroporator (MaxCyte, Rockville, MD, USA) with proprietary R-50×3 processing assemblies (MaxCyte). Prior to electroporation, 2.5 million cells were collected, washed once with PBS, twice with MaxCyte electroporation buffer, and finally resuspended in 50 μL of electroporation buffer. A total of 90 nmol/L of Alt-R *Streptococcus pyogenes* Cas9 nuclease v.3 (Integrated DNA Technologies (IDT), Coralville, IA, USA) and 180 nmol/L sgRNA (IDT) were incubated together for 15 min to form the ribonucleoprotein (RNP) complex. After transfection with Cas9 RNP or dsRed mRNA, cells were allowed to recover for 15 min in an incubator (5% CO_2_, 37°C) as described previously.^56^

**Table 1:** sgRNAs used in this study

sgRNA	Target	Sequence
sgRNA 1	*NUTM1* exon 2	TCAGGTTTGGGCACTAACTG
sgRNA 2	*NUTM1* exon 2	CCATCTGAAGCCATCCTCTC
sgRNA 3	*NUTM1* exon 3	TTCATGCTCATATCCGGTCC
sgRNA 4	*NUTM1* exon 3	CTGGTGGGTCAGAAGTTGGT
sgRNA 5	*NUTM1* exon 3	AGGGCCACATGGGACAACCG
sgRNA 6	*BRD4* exon 2	ACTAGCATGTCTGCGGAGAG
sgRNA 7	*BRD4* exon 2	GATTTCTCAATCTCGTCCCA
sgRNA 8	*BRD4* exon 2	TTCAGCTTGACGGCATCCAC

### Gene editing analysis

To detect insertions, deletions (indels) and knockouts (frameshift or indel >20 bp), DNA was extracted 72 hpt using the Blood and Cell Culture DNA Kit (QIAGEN, Hilden, Germany). Target regions were amplified by polymerase chain reaction (PCR) using NEB OneTaq 2X MM (Ipswich, MA, USA) and the corresponding primers (Eurofins Genomics, Ebersberg, Germany) listed in Table S2. PCR products were purified using a PCR Purification Kit (QIAGEN) and sequenced by Microsynth (Balgach, Switzerland) via Sanger sequencing (Figures S4 and S5). Indel and knockout scores were computed using Synthego’s ICE software.^57^^,^^58^

### MTS assay

After transfection, 5,000 cells per well were seeded in 96-well plates. After 24 h, MTS solution (Promega, Madison, WI, USA) was added to each well. Following a 2 h incubation period, optical absorbance was measured at 490 nm using a microplate reader.

### Flow cytometry

To quantify the fraction of cells transfected with dsRed mRNA, cells were brought into suspension 24 hpt and analyzed using BD FACSCalibur flow cytometer (BD Biosciences, San Diego, CA, USA). For detection of apoptosis, cells were harvested 72 hpt and stained with annexin V and propidium iodide (PI) from the Pacific Blue annexin V Apoptosis Detection Kit with PI (BioLegend, San Diego, CA, USA). Following 15 min of incubation, samples were run on the Attune NxT acoustic focusing cytometer (Thermo Fisher Scientific, Waltham, MA, USA).

For cell cycle analysis, cells were harvested 72 hpt. After washing and pelleting, 200,000 cells were resuspended in cold 70% ethanol for one hour for fixation. Following washing with PBS, cells were treated with 5 μg RNAse (Biozym Scientific, Hessisch Oldendorf, Germany) and 8 μg PI (Thermo Fisher Scientific) for 30 min. Samples were analyzed using the Attune NxT acoustic focusing cytometer (Thermo Fisher Scientific). All flow cytometry data were analyzed using FlowJo v.10 software (FlowJo, Ashland, OR, USA).

### Sulforhodamine B assay

After transfection, 1,000 cells per well were seeded in 96-well plates. At 96 hpt, cells were fixed using cold 10% trichloroacetic acid. Following washing and drying of the wells, the fixed cells were stained with 4% sulforhodamine B (SRB) in 1% acetic acid. The SRB solution was discarded, and the wells were washed three times with acetic acid. Once dried, the stained cellular proteins were resuspended in 10 mmol/L Tris (pH 10.5) and aliquoted into two wells per one original well. Optical absorbance was measured at 565 nm using a microplate reader.

### Colony formation assay

At 72 hpt, cells were counted and carefully seeded at 1,000 cells per well in 6-well plates, in DMEM + 10% FBS, thus ensuring even distribution of single cells in each well. After growing the cells for six days (143100) or eight days (HCC2429), cells were fixed and stained with 0.1% crystal violet containing 5% ethanol and 10% formaldehyde. For analysis, each well was photographed, and the number of colonies was counted using ImageJ.^59^

### xCELLigence real-time cell analysis

After transfection, cells were sieved using a 40 μm cell strainer and counted. A total of 5,000 cells per well were seeded in 96-well E-plates (Roche Applied Science, Penzberg, Germany). Electrical impedance was measured every 30 min using the xCELLigence RTCA SP System (Roche) and analyzed with RTCA software version 1.2.1 (Roche).

### Western blot

At the desired time point, cells were harvested by scraping and suspended in lysis buffer (containing 50 mmol/L Tris [pH 7.6], 150 mmol/L NaCl, 1% IGEPAL, complete proteinase inhibitor [Roche]). To obtain cell lysates, cells underwent three freeze-thaw cycles, followed by centrifugation. The protein concentration of the supernatant was measured using the Bradford assay (Bio-Rad, Hercules, CA, USA). For gel electrophoresis, 50 μg of protein from the cell lysate was supplemented with sodium dodecyl sulfate (SDS) gel-loading buffer containing 1% 2-mercaptoethanol and denatured by heating to 95°C for 5 min. Samples were run on a 6% SDS polyacrylamide gel electrophoresis and blotted onto a polyvinylidene fluoride membrane (Hybond-P, GE Healthcare, Waukesha, WI, USA) using a Mini Trans-Blot Cell (Bio-Rad).

Membranes were blocked with 5% non-fat dry milk in a mixture of tris-buffered saline and Polysorbate 20 (TBS-T) and incubated with primary antibodies targeting c-Myc (no. 9402, 1:500 dilution, Cell Signaling, Danvers, MA, USA), NUT (clone C52B1, no.3625, 1:1,000 dilution, Cell Signaling), and vinculin (1:5000 dilution, Sigma-Aldrich, Saint Louis, MO, USA) in TBS-T with 5% bovine serum albumin (BSA). Subsequently, membranes were incubated with horseradish peroxidase-conjugated antibodies targeting mouse or rabbit IgG (1:8000 dilution, Bio-Rad). Bands were detected using Amersham ECL Western Blotting Detection reagents (GE Healthcare) and visualized with the ChemiDoc MP Imaging System (Bio-Rad).

### Immunofluorescence microscopy

After transfection, cells were seeded in 24-well plates and grown for 72 h. Cells were then fixed with 4% formaldehyde, permeabilized with 0.1% Triton X-100 (Sigma-Aldrich, no. 93443) and blocked with 3% BSA. For cell size analysis, cells were incubated with anti-β-actin (1:100 dilution, clone AC-74, no. A2228, Sigma-Aldrich), followed by Alexa Fluor 488 (1:500 dilution, no. A11001, Thermo Fisher Scientific) as secondary antibody. Nuclei were stained with 2 μg/mL DAPI (Carl Roth, Karlsruhe, Germany). For visualization of NUT protein, cells were incubated with anti-NUT (1:500 dilution, clone C52B1, no. 3625, Cell Signaling) followed by Alexa Fluor 546 (1:250 dilution, no. A11010, Thermo Fisher Scientific) as secondary antibody. Cells treated with the BET inhibitor BI894999 (Boehringer Ingelheim, Ingelheim, Germany) were exposed to a 50 nmol/L solution for 30 min, followed by fixation.

For quantification of cell area and cytoplasmic to nuclear area ratios, images were acquired at 20× magnification and analyzed using CellProfiler 4.^60^ Nuclei were segmented from the DAPI channel using the IdentifyPrimaryObjects module and cytoplasmic boundaries were defined from the β-actin channel using the IdentifySecondaryObjects module (propagation method with adaptive thresholding, minimum cross-entropy). Clustered or incorrectly segmented cells were removed using the EditObjectsManually module. Cytoplasmic and nuclear areas were extracted with the MeasureObjectSizeShape module.

### Off-target analysis

Potential off-target sites were predicted using CasOFFinder.^61^ After electroporation with a CAS9 RNP carrying *NUTM1* ex3 sg3, DNA was isolated from HEK293 cells using the Blood and Cell Culture DNA Kit (QIAGEN) at 72 hpt and shipped to Novogene (Beijing, China) for whole genome sequencing (WGS) at 50× coverage. Reads were aligned to the hg38 reference genome using the Burrows-Wheeler Aligner software and sorted by chromosome position with Sambamba.^62^^,^^63^ Picard tools were used to merge BAM files and filter duplicate reads (http://broadinstitute.github.io/picard/). CRISPRessoWGS was used to identify indels within a 10 bp window surrounding the expected cut site of Cas9 at possible off-target sites, applying default parameters and a minimum number of one read per region for inclusion in the analysis.^64^ The error rate was calculated by dividing the number of bases inserted or deleted by the number of bases analyzed.

### Statistical analysis

Statistical analyses were performed with GraphPad Prism version 10 (GraphPad Software, San Diego, CA, USA). SRB, colony formation assays, xCELLigence real-time cell analysis, cell area and cytoplasm to nucleus ratios were evaluated by one-way ANOVA followed by Dunnett’s post-hoc test, using the electroporation control group as the reference. Cell-cycle arrest and apoptosis were analyzed using unpaired Student’s *t* tests. A significance level of *p* < 0.05 was used to reject the null hypothesis that there was no difference between the groups tested. We expressed the level of significance with the following annotations in the figures: ∗*p* < 0.05, ∗∗*p* < 0.01, ∗∗∗*p* < 0.005, and ∗∗∗∗*p* < 0.0001.

## Data and code availability

The data generated or analyzed during this study are available from the corresponding author upon request.

## Acknowledgments

We thank MaxCyte Inc. for providing the ExPERT GTx electroporator and Boehringer Ingelheim for supplying the BET inhibitor BI894999. We also acknowledge support from the Open Access Publishing Fund of the University of Tübingen. The graphical abstract and Figure 1A were created using BioRender.com. This study was funded by the 10.13039/501100001659German Research Foundation (DFG, project number 514598620), the intramural fortüne program of the 10.13039/501100009397Faculty of Medicine, University of Tübingen (proposal number 3007-0-0), and the University Children Hospital Tübingen.

## Author contributions

Conceptualization, U.M.L., M.M., and L.D.K.; formal analysis, M.F.C. and L.D.K.; funding acquisition, U.M.L., M.M., and L.D.K.; investigation, M.F.C. and T.M.G.; methodology, M.F.C., T.M.G., U.M.L., M.M., and L.D.K.; project administration, L.D.K.; resources, A.R.-M., U.M.L., and M.M.; software, M.F.C.; supervision, T.M.G., U.M.L., M.M., and L.D.K.; validation, U.M.L., M.M., and L.D.K.; visualization, M.F.C.; writing – original draft, M.F.C. and L.D.K.; writing – review & editing, T.M.G., J.S.A., A.R.-M., M.E.C., U.M.L., and M.M.

## Declaration of interests

A.R.-M. is an employee of MaxCyte Inc.

## Supplemental information

Document S1. Figures S1–S6, Tables S1 and S2

Document S2. Article plus supplemental information
