# Supplementary material for: Targeting oncogenic fusion-driven NUT carcinoma with CRISPR-Cas9 genome editing
Source: Mol Ther Oncol. 2025 Oct 6;33(4):201068. doi: 10.1016/j.omton.2025.201068 (PMC12581649; doi:10.1016/j.omton.2025.201068)
Supplement: Document S1. Figures S1–S6, Tables S1 and S2 [file mmc1.pdf]

OMTON, Volume *33*

Supplemental information

Targeting oncogenic fusion-driven NUT carcinoma with CRISPR-Cas9 genome editing

Maxim F. Carle, Tahereh Mohammadian Gol, Justin S. Antony, Alicia Roig-Merino, Mary E. Carter, Ulrich M. Lauer, Markus Mezger, and Linus D. Kloker

Table S1

Indels identified at predicted off-target sites. Base mismatches from sgRNAs are indicated in lowercase, deletions are highlighted in pink and insertions in blue. Likely off-target hits are highlighted in yellow. DP is the total number of reads spanning the specific position, DV is the number of reads supporting the variant.

Off-target sites for 3 mismatches to sgRNA:						
Position		Gene	Intron/Exon	Sequence	DP	DV
None						
Off-target sites for 4 mismatches to sgRNA:						
Position		Gene	Intron/Exon	Sequence	DP	DV
chr1	5503900	Intergenic		AGGGCCACAgGGGAaAgCaGGGG	48	48
chr3	11470707	ATG7	Intron	AGGGCCACAgGGGcCAcCaGAGG	36	1
chr5	127956637	SLC12A2-DT	Intron	AGGGCaACATGGGcCAggCGCGG	69	1
chr16	74919114	WDR59	Intron	AGGGCCcCtTGGGACcAgCGGGG	53	1
Off-target sites for 5 mismatches to sgRNA:						
Position		Gene	Intron/Exon	Sequence	DP	DV
chr1	23589021	MDS2	Intron	AGGGCCACATGGGgaAtCtcTGG	44	44
chr9	125994316	Intergenic		AGGagCAgATGGGACAgaCGGGG	41	2
chr10	6338928	Intergenic		AGGGCCACActGGgCAcCCcAGG	37	2
chr19	1472418	APC2	Intron	cGGcCCACATGGGgaAACaGAGG	55	2
chr1	56125191	ENSG00000297128	Intron	AGGcCCACATGGagCAAggGAGG	26	1
chr1	161395366	ENSG00000297047	Intron	tGGGCaACATGGcAaAACCcAGG	29	1
chr1	243449647	SDCCAG8	Intron	AGGGCCAtgTGGaAaAACtGGGG	78	1
chr2	232328722	DIS3L2	Intron	AGGGagACATGGGgCtAtCGGGG	35	1
chr5	94803823	MCTP1	Intron	AGGGagACATGGaAaAAtCGGGG	73	1
chr6	48912699	Intergenic		gtGGCCACATGaGACAAtgGGGG	50	1
chr6	168235516	ENSG00000228361	Intron	AGGagCACATGtGAtAAACCaGGG	11	1
chr7	1181505	ENSG00000307981	Intron	AGaGCCtCtgGGGACAACaGGGG	39	1
chr9	128601529	SPTAN1	Intron	tGGGCaACATGGcAaAACCcCGG	42	1
chr9	136629317	Intergenic		AGGGCCcCAgGGGcaAACCcAGG	42	1
chr9	137044099	NPDC1	Intron	AGGGCCACggGGGcCAcaCGGGG	36	1
chr9	137328697	EXD3	Intron	AGGGtCACAcGGGAGCtACacGGG	29	1
chr9	137328817	EXD3	Intron	AGGGtCACAcGGGAGCtACacGGG	22	1
chr11	110197086	RDX	Intron	tGGGCaACATGGcAaAACCcTGG	65	1
chr16	1254297	Intergenic		AGGGaCACAcGGGgacACCGGGG	43	1
chr16	35727184	Intergenic		AGaGaCACcTGGGAgAACtGAGG	33	1
chr16	75239969	BCAR1	Intron	AtGGtCAgATGGGACccCCGGGG	38	1
chr16	89670755	SPATA33	Exon (3'-UTR)	ctGGgCACATGGGACAgCCaTGG	60	1
chr18	25924934	ENSG00000295104	Intron	AGaGCCAtATtGGaAaAAtCGCGG	79	1
chr22	25404922	ENSG00000290796	Intron	cGGGCCACAcGGGcCcAgCGCGG	24	1
chr22	29084572	KREMEN1	Intron	AGaGCCAgATGGGAaAAgaGTGG	49	1
chrX	106606052	Intergenic		tGGGCaACATGGcAaAACCcAGG	54	1

Table S2

PCR primers used in this study.

Amplicon	Sequence
BRD4 exon 2 forward primer	TGCTGGCTCCTAGTGGGCTCTC
BRD4 exon 2 reverse primer	GGGCCTTCCTTTCTCCCACTGC
NUTM1 exon 2 forward primer	GCAGAATGCCTTGAGTTCCG
NUTM1 exon 2 reverse primer	AGCTCAAGCATGGCAAAATCC
NUTM1 exon 3 sgRNA 3-4 forward primer	TGGGGAATTCAGATGGCTAACTCTGG
NUTM1 exon 3 sgRNA 3-4 reverse primer	TCCGGGCCAAGGCTTTGTAACG
NUTM1 exon 3 sgRNA 5 forward primer	TGACAGCATCTAATGTGAAGACCA
NUTM1 exon 3 sgRNA 5 reverse primer	GGAAGTGGGTTAAGAGGCTGA

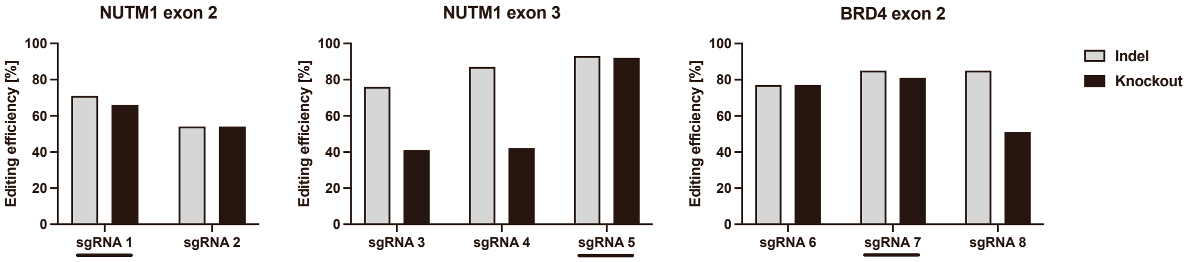

Figure S1

Comparison of indel and knockout efficacy of different sgRNA designs. A549 cells were transfected with Cas9 and different sgRNAs targeting *NUTM1* exon 2 and 3 and *BRD4* exon 2. DNA was extracted at 72 hpt to evaluate indel frequency and knockout scores using Sanger sequencing. The most effective sgRNAs used for subsequent experiments are underlined.

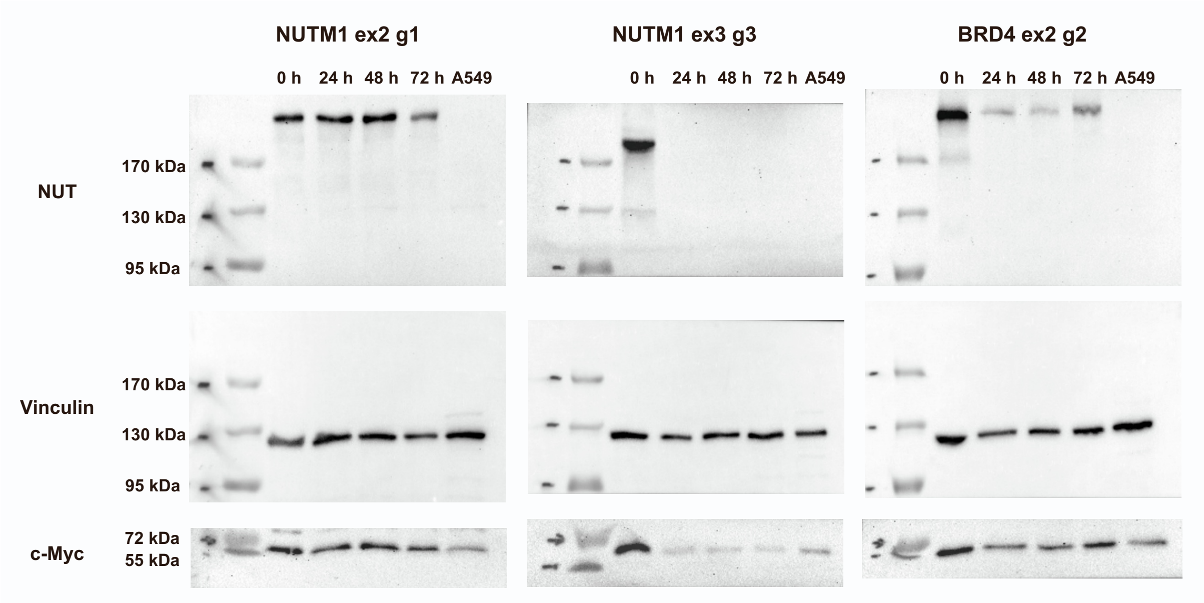

Figure S2

Uncropped Western Blots of NC cell line HCC2429 after transfection with Cas9 RNP and three different sgRNAs (*NUTM1* ex-2 sgRNA-1, *NUTM1* ex-3 sgRNA-5, *BRD4* ex-2 sgRNA-7).

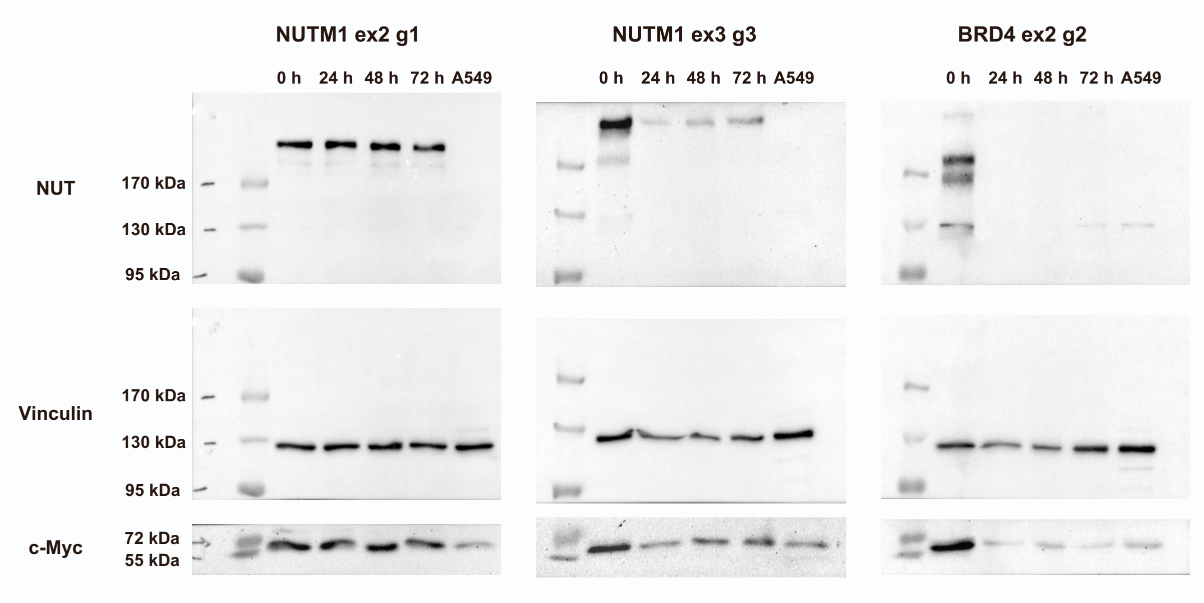

Figure S3

Uncropped Western Blots of NC cell line 143100 after transfection with Cas9 RNP and three different sgRNAs (*NUTM1* ex-2 sgRNA-1, *NUTM1* ex-3 sgRNA-5, *BRD4* ex-2 sgRNA-7).

NUTM1 ex-2 sgRNA-1:

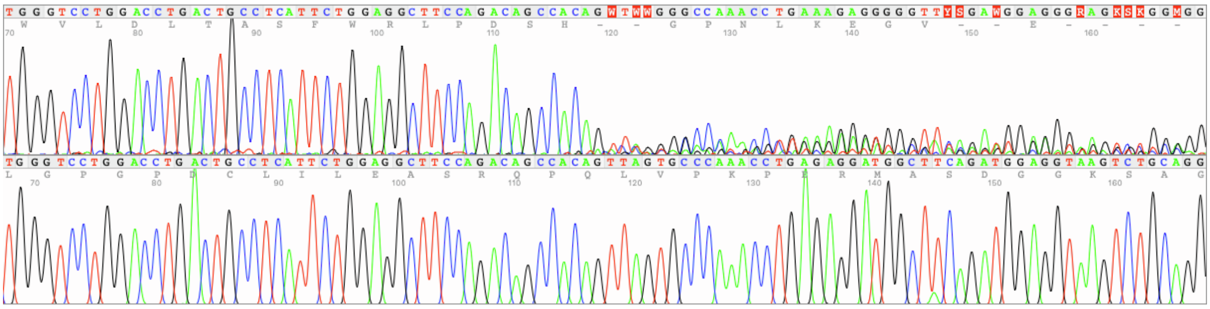

NUTM1 ex-3 sgRNA-5:

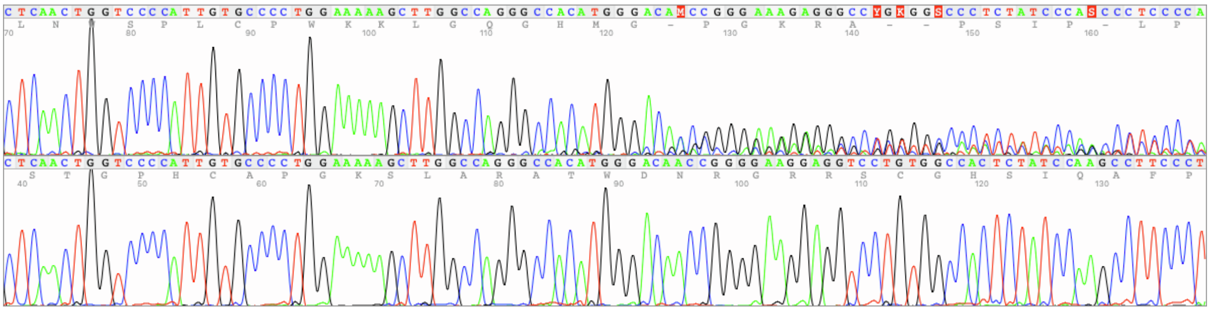

BRD4 ex-2 sgRNA-7:

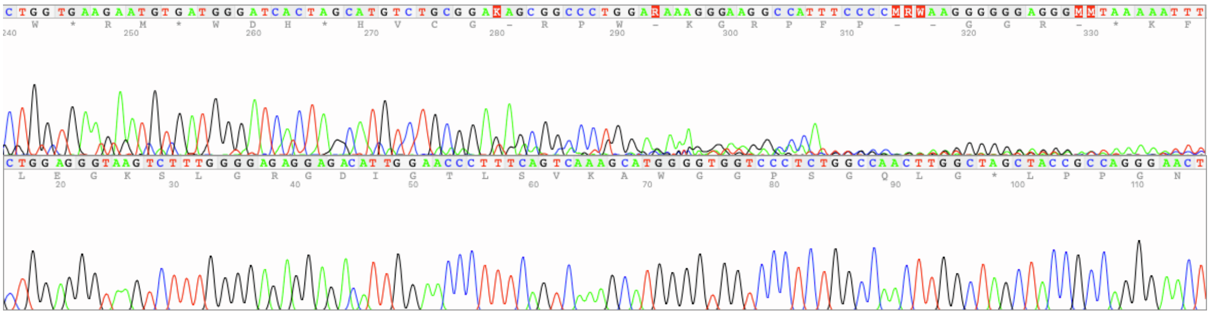

Figure S4

Representative images of Sanger sequencing from NC cell line HCC2429 after transfection with Cas9 RNP and three different sgRNAs (*NUTM1* ex-2 sgRNA-1, *NUTM1* ex-3 sgRNA-5, *BRD4* ex-2 sgRNA-7), compared to reference genome.

NUTM1 ex-2 sgRNA-1:

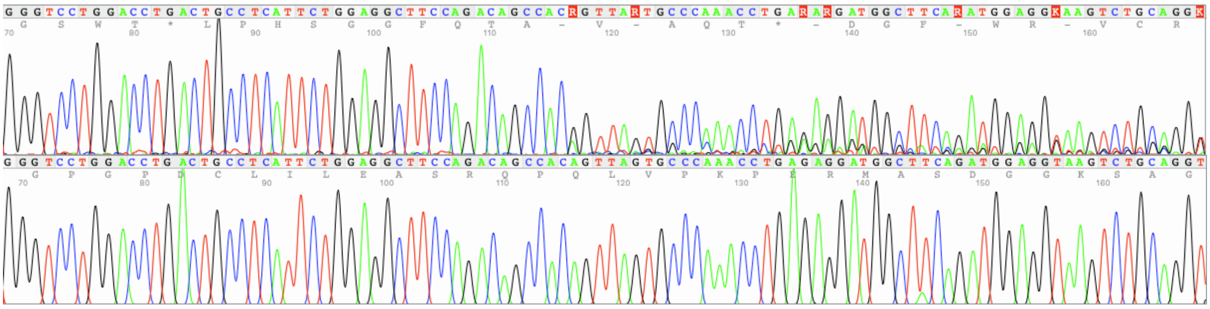

NUTM1 ex-3 sgRNA-5:

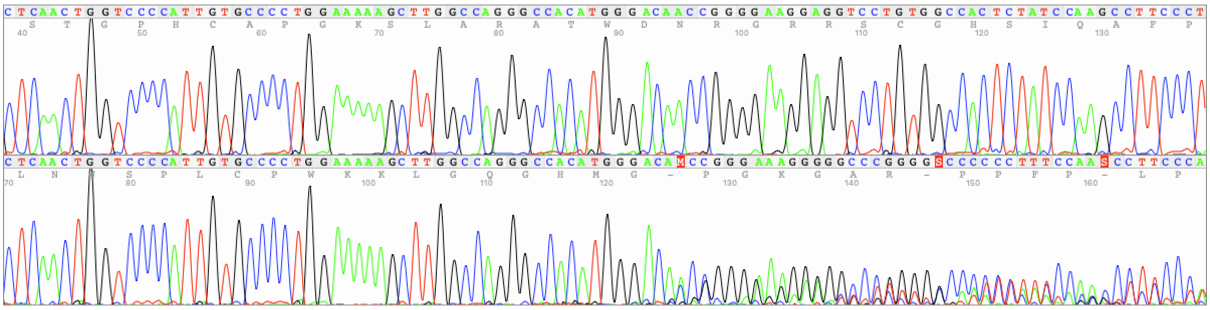

BRD4 ex-2 sgRNA-7:

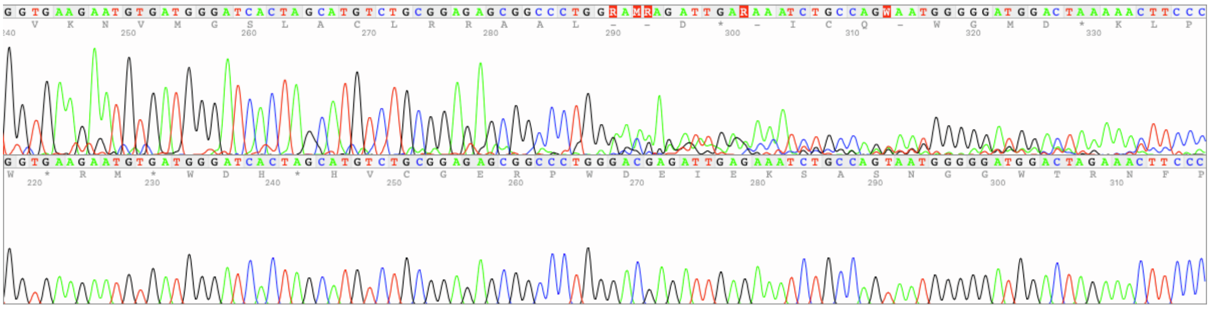

Figure S5

Representative images of Sanger sequencing from NC cell line 143100 after transfection with Cas9 RNP and three different sgRNAs (*NUTM1* ex-2 sgRNA-1, *NUTM1* ex-3 sgRNA-5, *BRD4* ex-2 sgRNA-7), compared to reference genome.

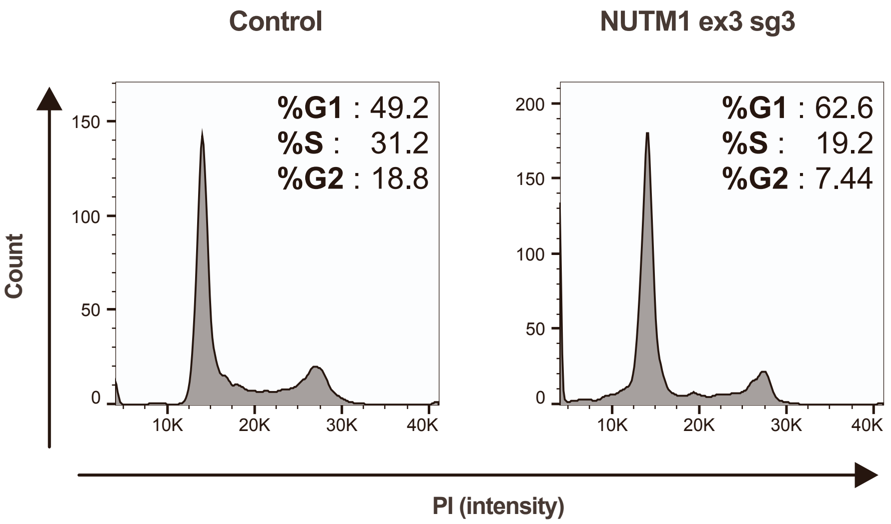

Figure S6

Cell cycle histogram of NC cell line HCC2429. Comparison of untreated (control) cells and cells 72 hpt with Cas9 RNP and NUTM1 ex-3 sgRNA-5. Fraction of cells in each phase of the cell cycle were measured using PI staining and flow cytometry.
